# Evolution of Electronic Circuits using Carbon Nanotube Composites

**DOI:** 10.1038/srep32197

**Published:** 2016-08-25

**Authors:** M. K. Massey, A. Kotsialos, D. Volpati, E. Vissol-Gaudin, C. Pearson, L. Bowen, B. Obara, D. A. Zeze, C. Groves, M. C. Petty

**Affiliations:** 1 School of Engineering and Computing Sciences, Durham University, South Road, Durham, DH1 3LE, UK; 2 São Carlos Institute of Physics, University of São Paulo-USP, PO Box 369, 13566-590, São Carlos, SP, Brazil; 3 Department of Physics, Durham University, South Road, Durham, DH1 3LE, UK.

## Abstract

Evolution-in-materio concerns the computer controlled manipulation of material systems using external stimuli to train or evolve the material to perform a useful function. In this paper we demonstrate the evolution of a disordered composite material, using voltages as the external stimuli, into a form where a simple computational problem can be solved. The material consists of single-walled carbon nanotubes suspended in liquid crystal; the nanotubes act as a conductive network, with the liquid crystal providing a host medium to allow the conductive network to reorganise when voltages are applied. We show that the application of electric fields under computer control results in a significant change in the material morphology, favouring the solution to a classification task.

The conventional method of manufacturing electronic systems is to design discrete components (resistors, capacitors, transistors) and to link them together to form an integrated circuit. In contrast, *evolution-in-materio* aims to emulate nature and use computer-controlled evolution to create information processors^1^,^2^,^3^. Disordered material is trained via external stimuli, usually electric fields, to perform a specific computational task^4^,^5^,^6^,^7^,^8^. Most research to-date has used the training phase to optimise the electrical connections to the material, as for example in field-programmable gate arrays (FPGAs), without affecting its morphology^4^,^5^,^6^,^7^,^8^. In this work, carbon nanotube thin films, forming complex electrical networks, are used to perform simple computation. Before training, the individual carbon nanotube bundles are randomly distributed and connections between the electrodes are sparse. During the training process the material undergoes a physical reorganisation as new electrical pathways are formed to perform the desired computation. Our results provide a link between the physical properties of a material and its evolution as a computing device. This is an important correlation that has been missing in this emerging interdisciplinary subject, where materials are usually considered to be black boxes.

The field of evolvable hardware explores the integration of systems and functions into existing silicon circuitry^9^. Devices such as FPGAs are popular due to the numerous connections within the chip that can be modified by the applied input signals^10^. FPGAs only offer a means of programming existing hardware. *Evolution-in-materio*, introduced by Miller^1^, is a fundamentally different approach to electronic systems design. However, identifying the materials for exploitation has been a major challenge. Harding and Miller showed that functions such as logic gates, tone discriminators and maze solving could be emulated within liquid crystal displays^3^,^4^,^5^,^6^. Using a similar methodology, we and others have demonstrated the operation of logic gates and solved a variety of computational problems using carbon nanotube/polymer composites^7^,^8^,^11^,^12^ and systems of interconnected metallic nanopartcles^13^. There is also interest in exploiting memristors in unconventional computing applications^14^,^15^. All these materials/devices may be considered as ‘static’. The application of electric fields does not significantly perturb their morphology and the process of evolution is essentially that of optimising the magnitudes and distribution of the applied voltages. Here, we introduce a dynamic material that expands both the science and scope of *evolution-in-materio* processors. We consider this to be an alternative to biological substances that have been used in evolutionary computation experiments^16^,^17^. We move beyond the black-box approach to materials to demonstrate a clear link between the dynamic microscopic structure of the computational material and its evolving ability to solve a problem.

## Results and Discussion

**Figure 1:**
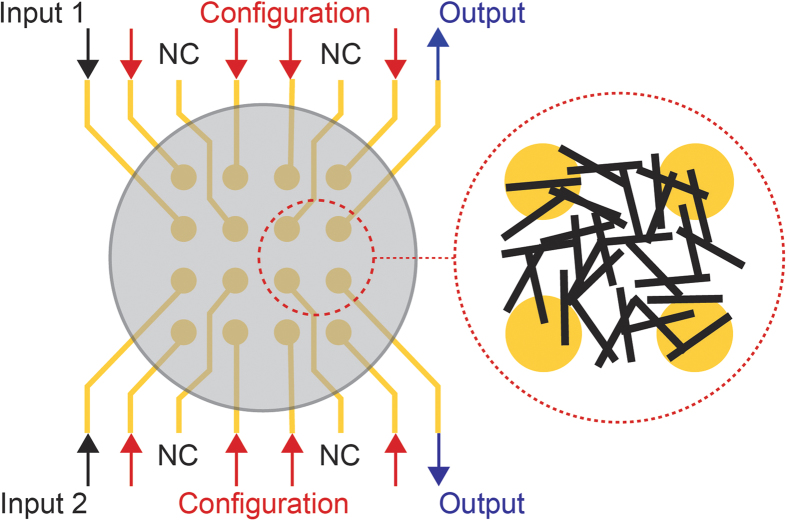
Evolution-in-materio of single-walled carbon nanotube/liquid crystal composites. Schematic diagram of the experiment with inputs, outputs and configuration signals to the micro-electrode array. NC = no connection.

Figure 1 depicts our experimental system. The diagram shows the 16 tracks and electrical contact pads (diameter of 50 μm and pitch of 100 μm) of a micro-electrode array. In the particular investigation described here, two of the contact pads were used as inputs, two as outputs and eight as configuration inputs (for training). The inputs, outputs and configuration inputs were all in the form of voltage signals. The magnified region shows four electrodes with a material (identified by the black bars) randomly distributed and forming a complex dynamic network of conductive pathways. The chosen material for this study is a composite based on conductive single-walled carbon nanotubes (SWCNTs) and insulating liquid crystals (LCs). The formulation and preparation techniques are given in the methods section. Previous work has shown that the nanotubes can interact and align with electric fields (in contrast to the previously studied nanotube/polymer network)^8^, hence they are well-suited for *evolution-in-materio* studies^18^.

**Figure 2:**
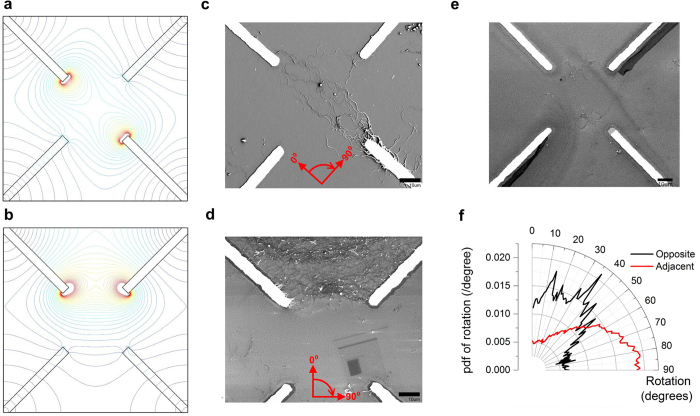
Alignment of single-walled carbon nanotubes within liquid crystal due to applied electric fields. Computer simulations (COMSOL) of electric field applied to; (**a)** Opposite electrodes. (**b)** Adjacent electrodes. SEM images showing alignment of SWCNTs with electric field applied to; (**c)** Opposite electrodes. (**d)** Adjacent electrodes. The axes relate to the orientation shown in (**f**). (**e**) No field applied. (**f)** Distribution of angles of SWCNTs in SEM images (**c**) + (**d**). Scale bars on SEM images are 10 µm.

Scanning electron microscopy (SEM) images for two different applied voltage conditions for a composite SWCNT/LC thin film are shown in Fig. 2. In each case, the material was deposited as described in the methods section and the images obtained following evaporation of the LC component; additionally a control sample was prepared with no applied bias. Computer simulations (using COMSOL) of nominally identical electrode arrays are provided in Fig. 2(a,b); the electric field magnitude is shown for the same configurations investigated in Fig. 2(c,d). It is common for SWCNTs to aggregate in solution;^19^ therefore the SEM images reveal aggregates or bundles of SWCNTs rather than individual nanotubes. The nanotube concentration is small in our experiments (0.0025% SWCNT by weight of LC) and the material is initially spread over a large area (∼3 mm diameter). Hence, as-deposited, only small bundles of SWCNTs will naturally be present in the central contact region (Fig. 2(e)). In the case of Fig. 2(c,d), a bias is applied (10 V for 15 min) between two of the electrodes, leaving the other two unconnected before evaporating the LC to reveal the SWCNT structure for SEM imaging. Two features are evident in these images. First, the density of nanotube bundles in the vicinity of the central electrode area is greater when a bias is applied; secondly, the orientation of the applied field influences the material morphology.

The distribution of electric field predicted in the simulations approximates the alignment of SWCNTs present in the experimental results. For example, with the bias applied to opposite electrodes, Fig. 2(c), the strong electric field region present between the electrodes (shown in the COMSOL simulation in Fig. 2(a)) results in an aligned nanotube network in the central contact area with very few connections to the unconnected electrodes. When the bias is applied to adjacent electrodes, Fig. 2(d), a very different morphology is evident. The electric field is concentrated between the top two electrodes (Fig. 2(b)), and as a result, the majority of material is attracted into this region. Image processing techniques were used to quantify the alignment shown in the SEM images^20^. Fig. 2(f) reveals the probability of each SWCNT bundle having a particular orientation. The nanotubes aligned using opposite electrodes show a trend to align along the axes of the electrodes, while for the adjacent case the alignment has changed by approximately 45°, agreeing with the predictions of the expected electric field patterns. However, the most significant observation from Fig. 2 is that the material can be manipulated by the applied electric field. Supplementary Information Figure 1 shows there is evidence of alignment in most samples studied; however, it should be noted that when extra material is present within the central contact area, the alignment process is obscured (Supplementary Information Figure 1(a,b)).

**Figure 3:**
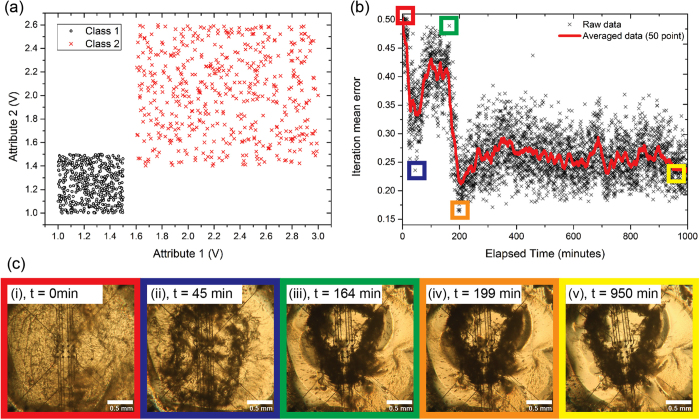
Evolution of a 2-class classifier using single-walled carbon nanotubes and liquid crystals as the computational material. (**a)** example set of training data with two distinct classes. (**b)** iteration mean error values for the classification problem versus elapsed time, including an average data line to clearly show the trend in the data. (**c)** optical micrographs (scale bars are 0.5 mm) show the electrodes and carbon nanotube/liquid crystal material at various different stages of evolution (from left to right 0 min; 45 min; 164 min; 199 min; 950 min respectively).

In order to test the ability of the SWCNT/LC composite to perform computation, a binary classification problem of separable data was used. The task involves detecting the class to which random inputs belong. The latter are in the form of voltage pulses (*x*_1_, *x*_2_) that belong to one of two possible classes. Figure 3(a) depicts the data and their clustering for the specific problem. This is a very simple task since it can be solved completely by identifying a threshold value 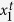 along the *x*_1_-axis and applying the rule “if 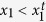 then (*x*_1_, *x*_2_) belongs to class 1, otherwise it belongs to class 2.” However, when using the material as an intermediate, this rule cannot be applied since the only means available are measurements (*O*_1_, *O*_2, …_*O*_*n*_) which is the combined response to (*x*_1_, *x*_2_). It is these measurements that inform the decision about the class to which the input (*x*_1_, *x*_2_) belongs. Based on an interpretation scheme utilising two output responses (O_1_, *O*_2_) to (*x*_1_, *x*_2_) an error can be defined and evaluated as discussed in the methods section. This allows an iterative population-based random search algorithm^21^ to be used for changing the local electrical properties of the material so that data classification results directly from a comparison operation of *O*_1_ and *O*_2_.

Figure 3(b) depicts the population-averaged error per iteration, while the corresponding minimum values are given in Supplementary Information Figure 2. A series of optical micrographs of the SWCNT/LC mixture are shown in (c) related to different stages of evolution. A video of this sequence is also provided as Supplementary Information. On the first iteration, the error value indicates a 50% probability of classifying a data point into the correct class. At this point the material is well dispersed and has not started forming new conductive pathways between the electrodes (micrograph (i)). For successive iterations the mean iteration error oscillates, reflecting a period where the material remains relatively static and the configuration voltages are adjusted by the algorithm to achieve the desired output. There is a subsequent decrease in the error up to *t *=* *45 min and an optimum value of 0.23 is achieved with relatively small changes in the structure (micrograph (ii)). Supplementary Information Figure 3 (where the population-averaged configuration voltages per iteration are shown) reveals that, during this period, the algorithm tries to exploit a local minimum. A dramatic change in the material structure (micrograph (iii)) causes the error value to temporarily rise to 0.48. In response, the algorithm moves away from the region where it has settled and tries to find a better solution. Between 164 min to 199 min the improved solution of 0.16 is found. The material morphology changes very little in this time (micrograph (iv)) and remains relatively unchanged thereafter indicating that a local minimum, (i.e. combined effect of material morphology and configuration voltages), has been reached. The material morphology retains its structure to the end of the experiment (micrograph (v)). Hence, the material has evolved under the control of the optimisation algorithm by the selective application of voltages to the thin-film network. This process has changed the morphology of the material to a state where its response has increased probabilities of providing the correct answer to the classification problem. Following system stabilisation, the remaining iteration mean error is approximately 0.25. We suggest that further work (different material systems and/or training algorithms) could lead to signifcantly smaller errors, even approaching zero.

In summary, we introduce liquid crystal/single-walled carbon nanotube thin film composites as a medium for evolution-in-materio studies. Changes to the morphology and micro-structure of the film were revealed by microscopy. These changes in the material micro-structural properties were directly correlated to the computational response of the material and the evolution of an elementary computing device. Following training, the dynamic response of the material system should be adequate for a number of real-time computational applications. An important question, which will be the subject of further study, concerns the reversibility of our nanotube network, i.e. what happens when the electric field is turned off? Our preliminary work suggests that there is some relaxation in the network, but that the initial material morphology, following deposition, is not regained. Many other materials, material architectures and training regimes (optical, magnetic, chemical) remain to be investigated. The work may lead to entirely new kinds of devices, either standalone structures or those that can be integrated with existing digital systems to provide more efficient and more effective signal processing architectures.

## Methods

### Material Formulation

Unsorted single-walled carbon nanotubes were obtained from Carbon Nanotechnologies Inc. (Houston, TX, USA) in dried powder form. The lengths vary from 100 nm to 1000 nm and diameters from 0.8 nm to 1.2 nm with the impurities being less than 15% (all values quoted from to manufacturers specifications). A commercially available liquid crystal blend (E7) was obtained from Merck Japan containing a mixture of four different liquid crystal molecules: 4-cyano-4′-n-pentyl-biphenyl (5CB), 4-cyano-4′-n-heptyl-biphenyl (7CB), 4-cyano-4′-n-oxyoctl-biphenyl (8OCB) and 4-cyano-4″-n-pentyl-p-terphenyl (5CT). Both materials were used as received, with no further purification. The SWCNTs were dispersed in the E7 by a short period of ultrasonication (Cole Palmer 750W ultrasonic homogenise, at 20% power for 1 min) followed by continuous agitation using a magnetic follower to keep the material dispersed between experiments.

### Electrode Fabrication

Electrodes were fabricated either on glass for the evolutionary training experiments, or silicon substrates for the SEM imaging. The silicon had an insulating 100 nm oxide layer to provide an electrically insulating support. In both cases, conventional etch-back photo-lithography was used to pattern electrodes in chromium/gold metal. The chromium seed layer had a thickness of 5 nm and the gold of 50 nm. An M3 nylon washer was attached to the electrode array with epoxy to contain the material whilst under test on the glass substrates for evolutionary experiments.

### Material Deposition

Material was prepared for SEM imaging by drop dispensing (∼1 μL) on silicon substrates. The array consisted of four electrodes of width 5 μm in a cross pattern with a central gap of 50 μm as shown in Fig. 2. The material was stimulated in two different configurations by applying a bias of 10 V for 15 min to opposite and to adjacent electrodes. A reference sample was also prepared where no bias was applied. For SEM studies the liquid crystal was evaporated by heating the substrate to 250 °C for 10 min on a hotplate, during which time the bias was continuously applied throughout the drying stage so any alignment caused by the electric field was maintained. A Keithley K2635a with custom Matlab software was used to apply the bias and measure the current. For evolutionary training experiments, material was drop dispensed (∼5 μL) into pre-prepared nylon washers glued onto glass substrates.

### SEM Imaging

The images were taken using an FEI Helios Nanolab 600 microscope and Hitachi SU70 microscope.

### COMSOL Simulations

Two-dimensional simulations of the electric fields present on the electrode arrays were created using COMSOL Multiphysics. Four metal electrodes were defined within an air domain. In each case, the simulation was set up with two electrodes floating and two electrodes with the bias applied between; one having a zero potential and the other a positive potential of 10. The lines of electric field were then shown on a contour plot for two cases: one with field applied between diagonally opposite electrodes and the other being between adjacent electrodes.

### Image Processing

To capture directional structures (ridges of valleys) in an image, a directional derivative-based approach has to be considered. In the 2D case, for a given pixel, the Hessian matrix was constructed by convolving the local image patch with the set of second order Gaussian derivative filters. Then, a direction of the ridge is given by the eigenvector with the largest eigenvalue of the Hessian matrix. Furthermore, to find ridges of various widths, a range of variances for the Gaussian was used and the most discriminant one was selected^20^.

### Electronics for Carrying Out Evolution

An mbed micro-controller was used to provide a serial interface to a PC where the computationally intensive algorithms could run. The mbed was responsible for controlling a series of analogue-to-digital and digital-to-analogue converters to provide signals and record the output of the material system. The inputs and outputs, in the form of voltage signals, were buffered so the high resistance material samples did not cause measurement errors.

### The Classification Problem

A simple binary classification based on highly separable data is presented here as an example of LC/SWCNT based *evolution-in-materio* computation. A two-dimensional process generates uniformly distributed data *x* = (*x*_1_, *x*_2_) that belong to either class *C*_1_ or class *C*_2_. When *x* ∈ *C*_*j*_, *j* = 1, 2, then 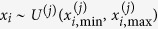, *i* = 1, 2. The task of the classifier in this case is to determine the class of an arbitrary random pair of data *x* generated by the process. Figure 3(a) depicts the data generated that are used for material training for *U*^(1)^(,) and *U*^(2)^(,). Two distinctive classes are shown, forming squares on the (*x*_1_, *x*_2_) plane. Thus, the problem of *evolution-in-materio* in this context is to evolve the LC/SWCNT material system into a state where its responses to a random input *x* can be uniquely interpreted to the correct class it belongs to.

A set of *K* observations *x*(*k*), *k* = 1, …, *K* with known classification *C*(*k*) = 1 or 2 was generated. Based on these, the following optimisation problem, with objective function *J*, is formulated for a particular individual *i* of a population of sise *N* used by an evolutionary algorithm with iteration index *I*.


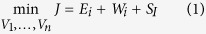



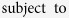



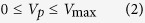


where 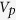, *p* = 1, …, *n* are the device’s configuration voltages, *E*(*k*) is the total error of the *in-materio* classification calculation when *x*^(*k*)^ is sent as input to the material and is given for individual *i* from


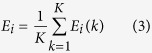


with


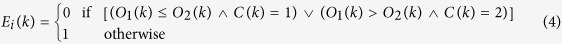


where *O*_1_(*k*) and *O*_2_(*k*) are measured voltages taken from two locations of the material’s body; *W*_*i*_ is a term penalising large voltages and is given by


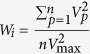


and *S*_*I*_ is an iteration quantity penalising non-responsive material states and is given by


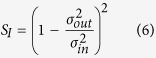


where 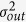 is the variance of the iteration’s *E*_*i*_, *i* = 1, …, 1 and 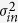 the variance of the population’s individual norms, i.e. the variance of the quantities 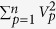 calculated for every individual. Equation (4) is a simple comparison scheme of the two measured voltages. The objective is to force the material to change its morphology and electrical behavior by use of an evolutionary optimisation algorithm so that the following interpretation scheme yields the correct results when a pair of data *x*(*k*) is given as input:


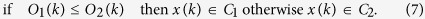


Problem (1), (2) is a nonlinear nonsmooth constrained optimisation problem with hardware in the loop. The evaluation of objective function (1) is performed based on the measured material responses *O*_1_ and *O*_2_. In view of the lack of any analytical and deterministic model of SWCNT/LC electrical properties, problem (1), (2) is addressed by population-based, derivative-free rather than gradient-based algorithms. Prior work^7^ showed that evolutionary algorithms are suitable for this task. A Differential Evolution (DE)^21^ was used here. Irrespective of the type of algorithm, the procedure for evaluating the objective function (1) is always the same. An individual in the population of the DE algorithm contains the information of the configuration voltages *V*_*p*_; the corresponding value of (3) is calculated by applying all *V*_*p*_ and holding these constant during the period while the *K* different pairs of data *x*(*k*) are sent as inputs to the material in the form of voltage pulses. For every *x*(*k*) the corresponding responses *O*_1_(*k*) and *O*_2_(*k*) are measured, allowing the application of (3) for the calculation of (1). This process is repeated within the optimisation algorithm whenever a call to the calculation of the objective function (1) is made. By following the iterative search pattern and actively minimising the classification error, the combination of configuration voltages and material morphology converge towards a system state that emulates a binary classifier.

## Additional Information

**How to cite this article**: Massey, M. K. *et al*. Evolution of Electronic Circuits using Carbon Nanotube Composites. *Sci. Rep.* **6**, 32197; doi: 10.1038/srep32197 (2016).

## Supplementary Material

Supplementary Video

Supplementary Information
