# Supplementary material for: Evolution of Electronic Circuits using Carbon Nanotube Composites
Source: Sci Rep. 2016 Aug 25;6:32197. doi: 10.1038/srep32197 (PMC4997311; doi:10.1038/srep32197)
Supplement: Supplementary Information [file srep32197-s2.pdf]

Evolution of Electronic Circuits using Carbon Nanotube Composites

M K Massey[1], A Kotsialos[1], D Volpati[2], E Vissol-Gaudin[1], C Pearson[1], L Bowen[3], B Obara[1], D A Zeze[1], C Groves[1], and M C Petty[1,*]

[1]School of Engineering and Computing Sciences, Durham University, South Road, Durham, DH1 3LE

[2]São Carlos Institute of Physics, University of São Paulo-USP, PO Box 369, 13566-590, São Carlos, SP, Brazil

[3]Department of Physics, Durham University, South Road, Durham, DH1 3LE

[*]m.c.petty@durham.ac.uk

Supplementary Information

List of Figures

1 **Additional SEM images.** Variation in alignment quality of SWCNTs between adjacent (top) electrodes (a + b) and opposite electrodes (c + d). Some alignment is evident in all samples, however when a larger amount of material has aggregated in the central area the alignment is less obvious (a + C). Scale bars are 10 µm . . . 2

2 **Iteration minimum error values.** Iteration minimum error values for the classification problem versus elapsed time, including an average data line to clearly show the trend in the data . . . 3

3 **Configuration voltage levels.** Population-averaged configuration voltages per iteration are shown for each configuration electrode. . . . 4

Additional Video

A video file is available online showing the data from Figure 3 in the main paper. On the left of the video is the sequence of optical micrographs synchronised with the graph to the right showing the iteration mean error values for the classification problem versus elapsed time, including an average data line to clearly show the trend in the data to the x-axis time scale on the right.

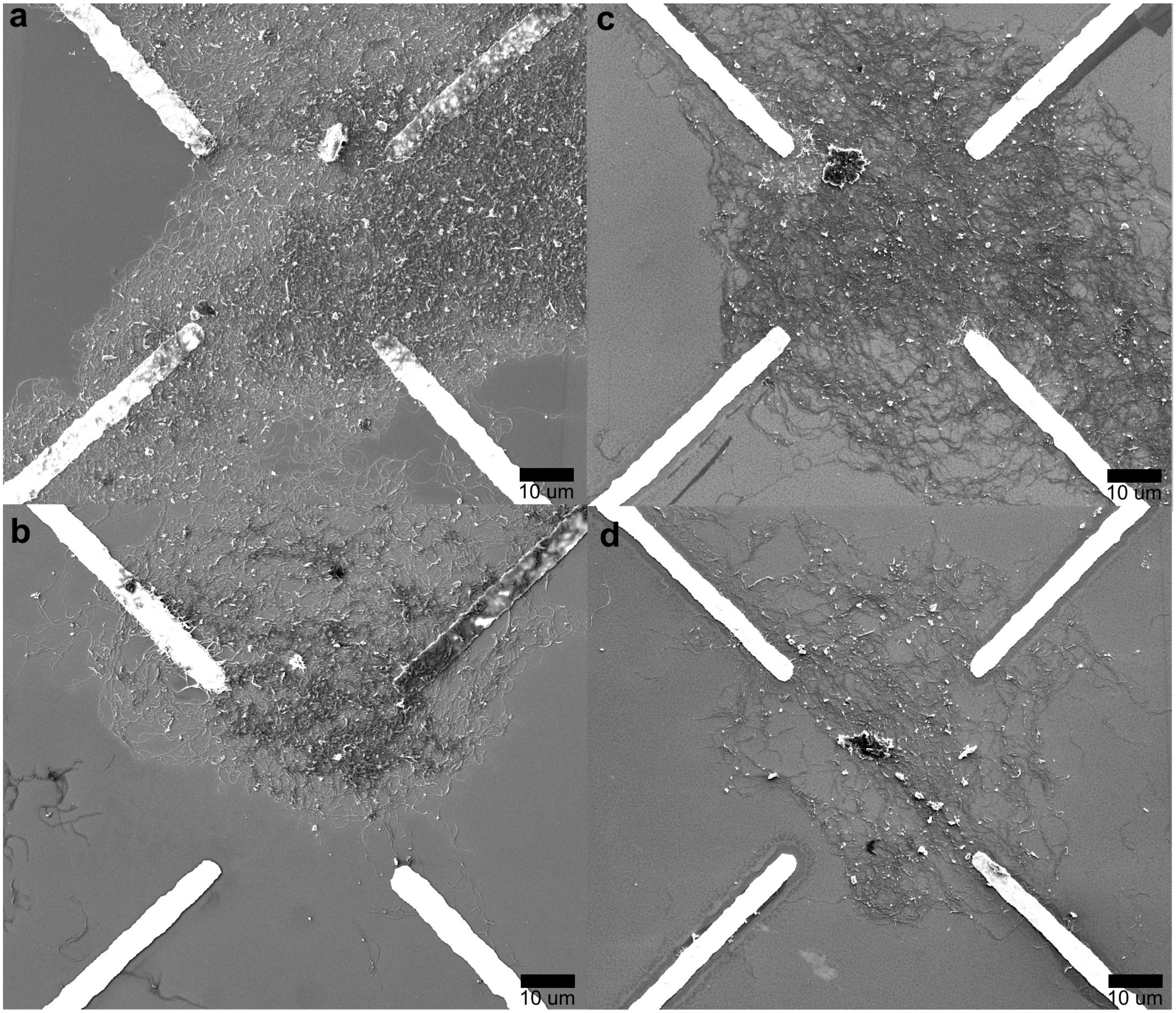

Figure 1. **Additional SEM images.** Variation in alignment quality of SWCNTs between adjacent (top) electrodes (a + b) and opposite electrodes (c + d). Some alignment is evident in all samples, however when a larger amount of material has aggregated in the central area the alignment is less obvious (a + C). Scale bars are 10 µm

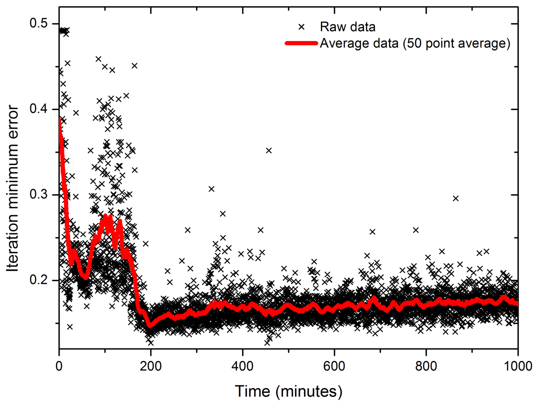

Figure 2. **Iteration minimum error values.** Iteration minimum error values for the classification problem versus elapsed time, including an average data line to clearly show the trend in the data

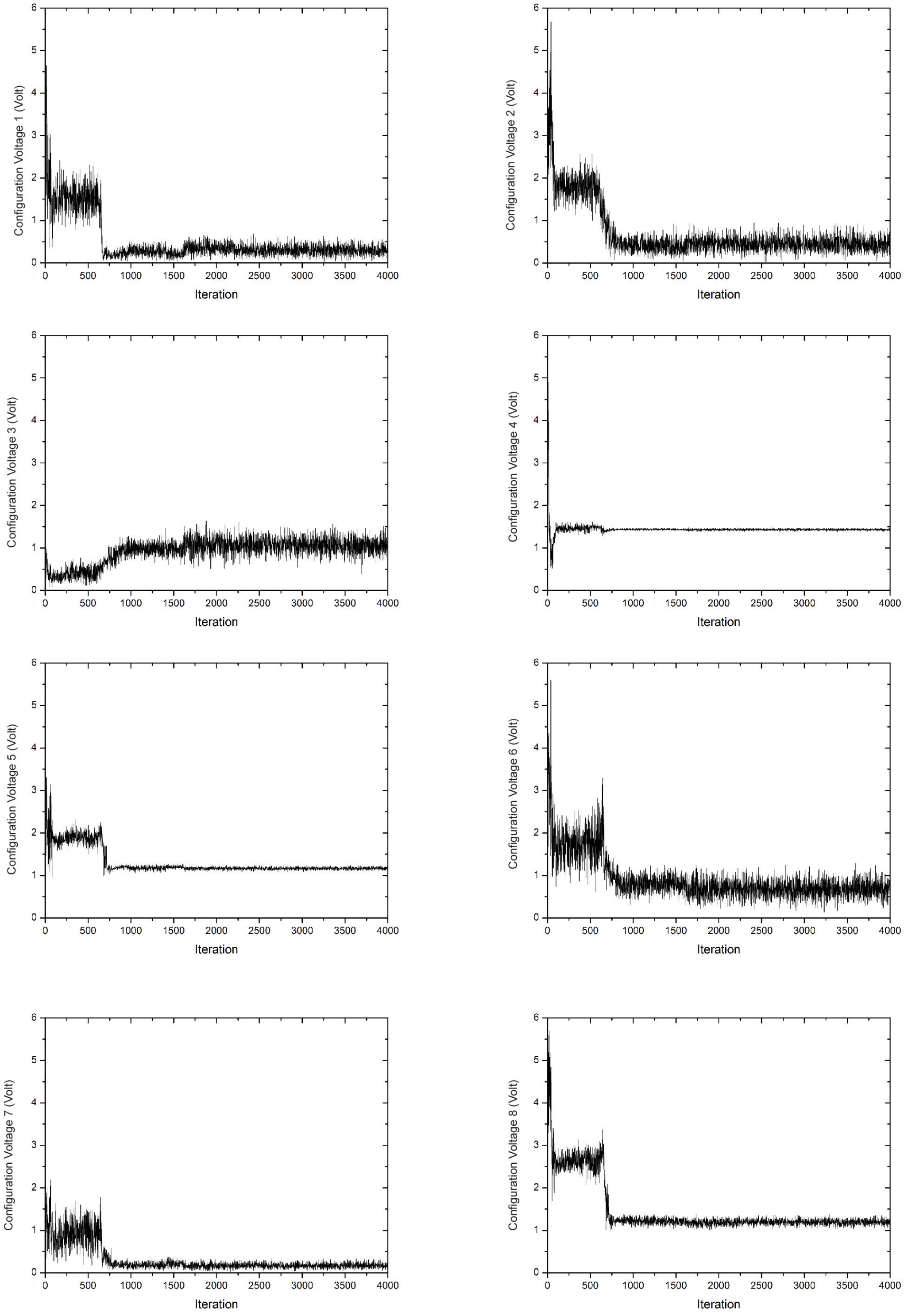

Figure 3. Configuration voltage levels. Population-averaged configuration voltages per iteration are shown for each configuration electrode.
